# Gender Dysphoria, Mental Health, and Poor Sleep Health Among Transgender and Gender Nonbinary Individuals: A Qualitative Study in New York City

**DOI:** 10.1089/trgh.2019.0007

**Published:** 2020-03-16

**Authors:** Salem Harry-Hernandez, Sari L. Reisner, Eric W. Schrimshaw, Asa Radix, Raiya Mallick, Denton Callander, Lili Suarez, Samuel Dubin, Aisha Khan, Dustin T. Duncan

**Affiliations:** ^1^ NYU Spatial Epidemiology Lab, Department of Population Health, NYU School of Medicine, New York, New York.; ^2^ Division of General Pediatrics, Boston Children's Hospital, Harvard Medical School, Boston, Massachusetts.; ^3^ Department of Epidemiology, Harvard T.H. Chan School of Public Health, Boston, Massachusetts.; ^4^ Department of Sociomedical Sciences, Mailman School of Public Health, Columbia University, New York, New York.; ^5^ Callen-Lorde Community Health Center, New York, New York.

**Keywords:** coping mechanisms, gender dysphoria, mental health, sleep health, transgender

## Abstract

**Background:** A vast amount of research has demonstrated the numerous adverse health risks of short sleep duration and poor sleep health among the general population, and increasing studies have been conducted among lesbian, gay, and bisexual individuals. However, although poor sleep health is disproportionately experienced by sexual and gender minority populations, little research has examined sleep quality and associated factors among transgender and gender nonbinary (TGNB) individuals. This study qualitatively explored the relationship that factors such as gender identity, mental health, and substance use have with sleep health among a sample of TGNB individuals in New York City.

**Methods:** Forty in-depth interviews were conducted among an ethnically diverse sample who identified as transgender male, transgender female, and gender nonbinary from July to August 2017. All interviews were transcribed, coded, and thematically analyzed for domains affecting overall sleep, including mental health, gender identity, and various coping mechanisms to improve overall sleep.

**Results:** TGNB interview participants frequently described one or more problems with sleeping. Some (15%) participants suggested that mental health issues caused them to have difficulty falling asleep, but that psychiatric medication was effective in reducing mental health issues and allowing them to sleep. An even larger number (35%) told us that their gender identity negatively impacted their sleep. Specifically, participants described that the presence of breasts, breast binding, stress and anxiety about their identity, and concerns about hormonal therapy and gender-affirming surgery were all reported as contributing to sleep problems. Given these sleep challenges, it is not surprising that most (60%) participants used various strategies to cope with and manage their sleep problems, including prescription and over-the-counter sleep medications (33%) and marijuana (18%).

**Conclusions:** Our findings document that sleep health is frequently an issue for TGNB individuals, and they also offer insight into the various ways that TGNB individuals attempt to cope with these sleep problems. Sleep health promotion interventions should be developed for TGNB people, which would promote positive mental health, reduce the risk of pharmaceutical adverse events, and help alleviate psychosocial stress in this target population.

## Introduction

Good sleep has been established in the literature as a critical element of overall health.^1^ Previous studies have documented the numerous adverse health outcomes associated with short sleep duration and poor sleep health of the general population, including increased risk of obesity, depression, anxiety, hypertension, stroke, mental illness, cardiovascular disease, diabetes, and other chronic health conditions.^2–12^ Moreover, a growing body of empirical research has demonstrated that poor sleep health is disproportionately experienced by sexual and gender minority populations.^13–26^

Increasingly, studies have focused on sleep among lesbian, gay, and bisexual (LGB) individuals, showing similar associations with adverse health outcomes.^16,18,19,25,27^ While evidence also strongly suggests that difficulties falling and remaining asleep may be risk factors for poor mental health, substance abuse, and sexual risk behaviors, most studies to-date have focused on gay, bisexual, and other men who have sex with men (MSM)^18,25,27,28^ with very little research conducted among other sexual minority groups and gender minority groups.

One study conducted in London surveyed 202 MSM, linking poor sleep quality and short sleep duration with increased depressive symptoms, substance abuse, and greater numbers of condomless sex partners.^18^ A recent Paris-based study of 559 MSM produced largely similar results, associating poor sleep with increased risk of substance abuse before and during sex as well as condomless intercourse.^25^ Lastly, a third study conducted in New York City analyzed screening data from 16,466 sexual minorities, which indicated links between sleep problems and poorer mental health, increased substance abuse, and worse HIV-related health outcomes.^17^ Therefore, while understudied, the existing literature points to significant health risks that may be unique to these populations.

Even markedly fewer sleep health studies have specifically looked at sleep disparities for gender minorities (i.e., transgender men, transgender women, and gender nonbinary individuals). This reflects a wider trend in research through which transgender and gender nonbinary (TGNB) individuals are often understudied or have been simplistically combined together with other minorities, which is problematic as it neglects the unique needs and experiences of these populations.

The only study to-date that has assessed sleep health disparities with respect to gender minorities was an analysis of sleep duration (very short: ≤5 hours; short: 6 hours; normal: 7–8 hours; and long: ≥9 hours per day) as recorded in the U.S. Behavioral Risk Factor Surveillance System.^16^ This study found that rates of very short sleep duration were highest among the 112 gender nonbinary individuals surveyed: 35.5% of transgender nonbinary participants reported short sleep, which compared with 17.3% of the overall lesbian, gay, bisexual, transgender (LGBT) respondents and 12.2% of cisgender straight respondents was the highest of any other group in that study.

To our knowledge, only two studies have examined sleep quality among TGNB individuals, with emerging evidence suggesting that some aspects of gender affirmation may have a contributory role in sleep quality. One study conducted in Germany analyzed data from 82 transgender women and 72 transgender men and found that poorer sleep quality was strongly associated with lower quality of life for both transgender women and transgender men when receiving hormone therapy versus no hormone therapy.^29^ However, another study argued that these effects are perhaps only slight, as administration of estrogen and antiandrogens in transgender women only had a small influence on sleep electroencephalogram, with an increase in the duration of shallow sleep.^30^

Taken together, the existing literature hints at disparities in sleep health among sexual and gender minorities while highlighting the need for further studies, especially for TGNB individuals. This need for further research is particularly important given that a number of factors disproportionately experienced by TGNB people (e.g., experiences of discrimination, employment challenges, financial concerns, and housing instability)^31^ could also play a part in sleep health.

No prior research, to our knowledge, has examined the perceived role of mental health, gender identity, and coping mechanisms on the sleep quality of TGNB individuals. Transgender sleep research needs further examination of a wide range of sleep characteristics to determine the cause and underlying mechanisms for poor sleep quality.

Qualitative methods in health research are useful for exploratory, underdeveloped areas of research and can deliver deeper understandings of understudied areas of transgender health, such as research that may help to isolate specific factors affecting sleep health for the transgender population. Qualitative methods can also provide important and unanticipated insights to the experiences of understudied populations such as transgender individuals by facilitating in-person interviews that allow for the unfolding of unique narratives. As such, the purpose of this study was to use qualitative interviews to examine how sleep health can impact health outcomes among TGNB individuals in New York City.

## Methods

### Participants and recruitment

Forty in-depth interviews were conducted from July to August 2017 with ethnically diverse TGNB-identified participants. Eligibility entailed participants who (1) were 18 or older, (2) could speak English, (3) identified as a gender identity that differed from the sex assigned at birth, (4) resided in one of the five boroughs of New York City (Manhattan, Bronx, Brooklyn, Queens, or Staten Island), and (5) were willing to be interviewed for approximately an hour. Assigned sex at birth (male/female), regardless of current gender identity, was used as a measure to balance the participants selected for the research study.

Recruitment occurred through personal networks, community organizations serving the LGB and TGNB communities, digital message boards, social networking sites (such as Facebook and Craigslist), and through word-of-mouth. In addition, recruitment letters and online flyers were sent electronically to social media groups with a predominantly TGNB audience. Such recruitment materials contained basic information about the nature of the research study, expected participant role in the study, and contact information. Eligibility screeners were conducted over the phone or over e-mail. Then, if eligible and willing to participate in the study, participants were scheduled a one-on-one in-depth interview at NYU Spatial Epidemiology Lab's office in the Manhattan borough of New York City.

Interview duration varied according to how much the participants were willing to disclose their experiences and daily lives. For verification, participants completed an eligibility screener in person before the in-person consent process. In total, only one participant was deemed ineligible and was not invited to participate in the study. The analytic sample consisted of 20 assigned males at birth and 20 assigned females at birth who completed interviews.

### Community involvement

TGNB-identified community member researchers were involved as consultants throughout multiple stages in study preparation and implementation, such as contributing to project design, providing feedback on interview guide content, and protocol details. This practice followed in-line with Participatory Action Research (PAR), which values participation by those affected by the study both throughout the research process and for putting research findings into action.^32–37^

Consultants were provided project materials for review. After review, consultants and research associates met for a 2-h meeting where consultants provided feedback, specifically focusing on refining the relevance of interview guide content and optimizing a culturally sensitive and legible delivery of interview questions. Consultants were reimbursed $200 cash at the end of the meeting and invited to continue to serve as a member of the investigative team for the remainder of the study. Consultants also returned once the interviews were complete to review the results and participate in data analysis.

### Procedure

Research assistants were trained by the investigative team in qualitative interview methods, including interview structuring, question types, and qualitative data analysis, and were supervised by PhD- and MD-level researchers with expertise in sexual and gender minority health. The research team developed the interview guide based on the background literature and modified it in collaboration with TGNB community consultants and the qualitative research method expert coinvestigators.

Before the interview, enrolled participants completed a short demographic survey. The survey gathered participant age, current gender identity, sex assigned at birth on original birth certificate, sexual orientation, race/ethnicity, New York City borough of residence, relationship status, level of education, employment status, sources of income in the past 12 months, total income the previous year, medical insurance, duration of time lived at their current primary address, U.S. residential ZIP code, number of times they moved in the last year, number and relationship to the people they live with, and type of location they had inhabited during the last year (e.g., emergency shelter, apartment, hospital, substance abuse treatment facility).

Although the interviewers had a semistructured guide, the flow and topics of discussion, along with the direction of the interviews, were modified based on the interviewee responses and sensitivity, thus allowing the interviewers to explore particular topics and the interviewee to express their unique narrative. Conducting interviews was equally divided even among three research assistants. Interviews ranged from 16 to 140 minutes in length (median 66.5, mean 70.08). Following the interview, participants were paid $50 in cash.

To ensure confidentiality, information provided from eligibility screeners, demographic surveys, and interviews were anonymized and proper nouns, names, and places were removed. Identification numbers were randomly assigned to each participant. All interviews were audio recorded and then transcribed verbatim for analysis. Audio recordings were destroyed once transcription was finalized.

### Ethical considerations

The Institutional Review Board at New York University School of Medicine approved the research study. All participants signed consent forms before participating. No adverse events were reported to investigators.

### Interview guide

The interview guide included questions to assess overall sleep, such as how participants would describe their sleep during the past month, if they do anything to help themselves go to sleep, and if their gender identity affects their sleep. Complete sleep health questions were as follows:

- During the past month, how would you describe your sleep overall?
- ○ What about your sleep makes you say that it is good/bad? (Why do you describe it as …?)
- ○ In what ways, if any, do you think that would change if you lived somewhere else?
- What about your neighborhood makes it easier to go to sleep?
- What about your neighborhood makes it more difficult to go to sleep?
- Do you do anything to help yourself go to sleep?
- Are there any medications or supplements that you take to help yourself sleep?
- Is there anything about your gender identity that you think affects your quality of sleep?

### Data analysis

All interviews were coded and thematically analyzed for domains affecting overall sleep.^38^ Our approach to the qualitative analysis followed several steps. First, the research assistants (who conducted the interviews) read the transcribed interviews. Then, they developed codes based on preliminary readings of the interview contents. Second, preliminary (broad) codes were generated by constructing a list of major codes derived from interview memory. These codes were then cross-checked among the team to finalize the code list.

The codes were then further refined with the help of research assistants who conducted the qualitative analyses. This process involved a random sample of interviews and developing specific codes. Cross-checking was conducted among a new batch of research assistants (who solely focused on data analysis), which included holding a meeting to agree on the codes. This approach resulted in one unified code list or codebook.

The generation of a final code list was conducted by the investigators on the project who have expertise in qualitative methods and/or LGBT health, and, especially, transgender health. The investigators received two randomly selected interviews for review for the finalization of codes. Once a final codebook was completed, inter-researcher reliability was assessed, which was assessed by applying the codes to five randomly selected transcripts, which were each then coded by two research assistants. The similarities and differences were discussed among the team to ascertain uniformity in extracting data for each code.

In the next step, transcripts were individually read by the research assistants, who then extracted excerpts for each code into separate files. To ensure that no relevant data were lost from analysis, excerpts were coded multiple times, especially in places where the research participant addressed more than one thing in a particular conversation. All extracted excerpts were labeled with the ID number of the transcript.

Next, for each code file, every excerpt was reread and summarized as notes. Each note represented a single idea, statement, or incident that emerged during analysis and coding. We took special care to use the interviewee's own words as much as possible, thereby retaining key words, which were then used to formulate themes.

Lastly, to generate themes, once the codes were finalized, research assistants then went back and applied fragments of interviewee responses to the codes. Codes with at least four different transcripts constituted as a theme. Each theme included the transcript ID numbers to identify where the idea was mentioned, and to establish those ideas that were more popular than others.^38^ The most popular responses related to sleep were chosen as themes for this study. In addition, each interviewee response was labeled with the participant's demographic information, including current gender identity (e.g., nonbinary, transgender male, transgender female), sexual orientation, and race/ethnicity.

## Results

### Sample characteristics

For the total sample, the majority of participants were either 18–21 years old (25%) or 30–33 years old (22.5%). In addition, most participants either identified as white (45%), black or African American (20%), or Hispanic or Latinx (30%). The majority of participants identified their current gender identity as transgender man (30%), transgender woman (27.5%), or gender nonbinary (35%). Forty-five percent of participants identified their sexual orientation as queer, and the majority of participants identified their relationship status as single (52.5%). Most participants reported earning under $10,000 (47.5%), and the majority either graduated from college (20%) or had some college, vocational school, or apprenticeship experience (37.5%). Complete sample characteristics are presented in Table 1.

**Table 1.** Sample Demographics (*n*=40)

	Assigned male (*n*=20)	Assigned female (*n*=20)	Overall (*n*=40)
Age			
18–21 Years old	15.0 (3)	35.0 (7)	25.0 (10)
22–25 Years old	10.0 (2)	15.0 (3)	12.5 (5)
26–29 Years old	10.0 (2)	20.0 (4)	15.0 (6)
30–33 Years old	25.0 (5)	20.0 (4)	22.5 (9)
34–37 Years old	15.0 (3)	5.0 (1)	10.0 (4)
38 Years and older	25.0 (5)	5.0 (1)	15.0 (6)
Current gender identity			
Female	55.0 (11)	5.0 (1)	30.0 (12)
Male		30.0 (6)	15.0 (6)
Transgender male, trans man, FTM	5.0 (1)	55.0 (11)	30.0 (12)
Transgender female, trans woman, MTF	55.0 (11)		27.5 (11)
Genderqueer, nonbinary, gender nonbinary	20.0 (4)	50.0 (10)	35.0 (14)
Other	5.0 (1)	15.0 (3)	10.0 (4)
Sexual orientation			
Lesbian, gay, homosexual	10.0 (2)	15.0 (3)	12.5 (5)
Straight, heterosexual	45.0 (9)	20.0 (4)	32.5 (13)
Bisexual	25.0 (5)	10.0 (2)	17.5 (7)
Queer	20.0 (4)	70.0 (14)	45.0 (18)
Don't know, not sure	5.0 (1)	10.0 (2)	7.5 (3)
Something else	5.0 (1)	5.0 (1)	5 (2)
Race/ethnicity			
White	45.0 (9)	45.0 (9)	45.0 (18)
Black or African American	25.0 (5)	15.0 (3)	20.0 (8)
Hispanic or Latino	30.0 (6)	30.0 (6)	30.0 (12)
Asian or Pacific Islander		10.0 (2)	5.0 (2)
Native American or Alaska Native	5.0 (1)		2.5 (1)
Biracial or multiracial	5.0 (1)	25.0 (5)	15.0 (6)
Educational attainment			
Less than 12th grade	25.0 (5)	10.0 (2)	17.5 (7)
High school degree, or equivalent	15.0 (3)	5.0 (1)	10.0 (4)
Some college, vocational school, or apprenticeship	40.0 (8)	35.0 (7)	37.5 (15)
Bachelor's degree	10.0 (2)	30.0 (6)	20.0 (8)
Graduate degree or higher	10.0 (2)	20.0 (4)	15.0 (6)
Employment status			
Employed full-time	20.0 (4)	30.0 (6)	25.0 (10)
Employed part-time	20. (4)	35.0 (7)	27.5 (11)
Unemployed	40.0 (8)	20.0 (4)	30.0 (12)
Student	15.0 (3)	35.0 (7)	25.0 (10)
Retired	5.0 (1)		2.5 (1)
Unable to work	10.0 (2)		5.0 (2)
Income			
Under $10,000	50.0 (10)	45.0 (9)	47.5 (19)
$10,000 to $24,999	25.0 (5)	35.0 (7)	30.0 (12)
$25,000 to $39,999	20.0 (4)	5.0 (1)	12.5 (5)
$40,000 or more	5.0 (1)	15.0 (3)	10.0 (4)
Health insurance coverage			
Yes	100.0 (20)	100.0 (20)	100.0 (40)
No			
Borough of residence			
Bronx	10.0 (2)	10.0 (2)	10.0 (4)
Brooklyn	40.0 (8)	65.0 (13)	52.5 (21)
Manhattan	40.0 (8)	15.0 (3)	27.5 (11)
Queens	5.0 (1)	10.0 (2)	7.5 (3)
Staten Island	5.0 (1)		2.5 (1)

FTM, female-to-male; MTF, male-to-female.

### Qualitative results

A substantial number of participants reported in their qualitative interviews that they experienced one or more sleep problems, including difficulties falling asleep, difficulties staying asleep, or an insufficient number of hours of sleep. Participants described a number of different factors that they believed contributed to their sleep problems, including some directly related to their gender identity. In addition, they also describe the ways they have attempted to cope with or manage their sleep problems. Below we describe the issues related to sleep problems described by our participants.

### Mental health

Six participants (15%) shared struggles with anxiety and/or depression that affected their overall sleep, including the use of various pharmaceuticals to treat mental illness and regulate sleep health:

My anxiety level has prevented me from getting to sleep easily, and during recent months, I've been able to adapt to new psychiatric medication that allows me to have less anxiety, as well as increasing that medication just before I go to bed. (Transgender female/non-binary, pansexual, White, age 34)

I will sometimes not be able to fall asleep because of anxiety, or I'll wake up in the morning earlier than I need to, and spiral into anxious thoughts that make it very difficult for me to fall back asleep again. I'll have stress dreams, or nightmares, even, that disrupt my sleep. (Non-binary/transfemme, queer, White, age 33)

I was diagnosed with depression. Before a month ago, I wasn't sleeping at all. There'll be days that I would stay up all day and then sleep all night, or sleep all day and be up all night, until I was prescribed antidepressants, and then that's what helped me go back into my sleep cycle. (Non-binary, gay/lesbian, Hispanic/Latino, age 31)

In addition, one participant described lack of quality sleep leading to poor eating habits such as overeating:

I have anxiety and I have depression and I take Meds for them but not sleeping well doesn't help. I get more anxious and don't feel so great, like mentally. I over-eat, especially when I sleep less I eat a lot more and I make bad choices about the kind of food that I eat. (Non-binary, queer, White, age 28)

The experiences shared indicate that the presence of mental health disorders, such as anxiety and depression, among TGNB individuals can contribute to disruptive sleep and possibly habits of overeating.

### Gender identity

Participants (*n*=14, 35%) described how their gender identity affected their sleep quality in different ways. Five participants (12.5%) reported that their physical bodies, particularly the presence of breasts, contributed to disruptive sleep:

I still sleep with a stuffed animal … I used to do that, in part, because I was shielding my arms from my chest. I didn't want to touch my chest in my sleep and be aware of things that I didn't want to be reminded of. Whereas I've had top surgery, so I don't have anything to be hitting all night and worried about. (Transgender male, queer, White, age 24)

I actually feel better not wearing a shirt when I sleep…there's something about it that's more freeing, but when I'm next to somebody, I'm very aware that I have breasts, and it's just—it makes me feel very self-conscious. (Non-binary and transmasculine, queer and pansexual, Asian/Pacific Islander, age 23)

One participant described breast binding (the act of flattening breasts by use of constrictive materials) as a coping mechanism in response to feelings of gender dysphoria:

I think that sometimes feelings of dysphoria can be heightened, especially—I bind. When I'm home and I'm not binding. I don't wear my binder to sleep, obviously. Though, I'm sure some people do that. I really shouldn't … That can make me more—I feel like my body is more visible. (Transgender male and non-binary, gay/lesbian, and queer, White, age 19)

Furthermore, six participants (15%) specifically discussed how their own gender self-identification, perception by society, and physical presentation impacted their sleep quality:

Identifying as non-binary and presenting as very binary is not comfortable for me. When I had less pressing things affecting my sleep, I would think about that more and I kinda stress about that more. (Non-binary, queer, White, age 28)

The perception of my body at any given time, and how severe my dysphoria may be. The stress that I encounter as a result of my gender identity outside, may be difficult for me to process, and may linger for longer than I would like. (Transgender female and non-binary, something else, White, age 34)

Anxiety that I have in my life stemming from my gender identity would be part of that. Then just having that kind of thought process in my head, having it be difficult to go to sleep. (Transgender male, queer, White, age 33)

Being trans, you always have a higher level of anxiety because you're constantly thinking about your gender. In general, that could cause you to have more trouble sleeping. (Transgender male and non-binary, queer, White, age 31)

Three participants commented on the role gender transition, specifically hormonal therapy and gender-affirming surgery, in their overall sleep health:

Before I was on hormones or before I had top surgery, I felt super stressed about my body all the time, and so that certainly—that stress level impacted things, but these days, I don't—I think it's pretty nonimpactful. (Transgender male, queer, White, age 29)

I'm on hormonal therapy and that makes—a day or two after I take my shot I get very sleepy. I sleep a lot then. (Transgender female, heterosexual, Black, age 26)

I had bottom surgery and it didn't come out as it was supposed. I'm facing more surgery. That keeps me up for a while. (Transgender male, heterosexual, Hispanic/Latino, age 60)

### Coping mechanisms

Participants (*n*=24, 60%) described various methods used to facilitate falling and staying asleep as well as to improve overall sleep hygiene. About one-third (*n*=13, 32.5%) shared that they have used or currently use either prescription or over-the-counter (OTC) sleep aid medication:

I'll take a Klonopin. I'm heavy into pills, I'll be honest with you. I'll take a pill, and I'll down, and I just wanna sleep—sleep it away. (Woman, heterosexual, biracial, age 48)

I take Lexapro and it helps me sleep, so I take it before bed. (Non-binary, queer, White, age 28)

I take Prazosin, which is a low blood pressure medication. There are studies that it treats nightmares. (Transgender male/non-binary, gay/lesbian and queer, White, age 19)

I used to take Seroquel, but, I stopped that for a while. Then they put me on Ambien. I would just rather go to sleep natural. (Transgender male, heterosexual, Hispanic/Latino, age 60)

I take sleeping pills sometimes. Triazolam. (Woman, don't know/not sure Hispanic/Latino, age 58)

I used to take Benadryl. It works but I still wake up at 3:00 in the morning. (Woman, bisexual, Black, age 30)

I take melatonin at night. Once in a while, if I'm really having a stretch where I'm having issues sleeping, I will obtain a pharmaceutical to help me sleep. (Transgender female, queer, White, Native American/Alaska Native, age 36)

In the effort to have a good night sleep, seven participants (17.5%) recounted inconsistently using marijuana for sleep aid purposes, and four participants described avoiding the use of cell phone or computer screens before bed:

I smoke weed sometimes, but other than that, usually I just lay down and just try to calm myself till I fall asleep or I just work on art or whatever till I'm exhausted. (Transgender female, bisexual, White, age 31)

I try to listen to relaxing things sometimes, just give myself a good amount of time away from computer screens. (Non-binary, queer, Hispanic/Latino, age 21)

If I avoid looking at my phone or playing on the computer, that's generally helpful for me to go to sleep, if I make myself do that. (Transgender male and non-binary, queer, White, age 31)

## Discussion

Overall, sleep health among TGNB individuals is an understudied area in need of further investigation. Because existing gender minority sleep research has been limited and has only utilized quantitative methods among transgender individuals,^29,30^ there is a need to identify unanticipated factors contributing to poor sleep among not only transgender but also gender nonbinary individuals through a qualitative approach. This is the first study, to the best of our knowledge, to examine the perceived role of mental health, gender identity, and coping mechanisms on the sleep quality of TGNB individuals through qualitative means. As such, our study serves to (1) identify factors that impact sleep health in a TGNB sample in New York City and (2) provide direction for future studies and intervention strategies.

Our analysis makes several contributions to the literature. First, one recent study discussed the heterogeneity in sleep deprivation and chronic health conditions by distinct LGBT groups, emphasizing the need to identify risk factors among sexual minorities within each group (lesbian vs. gay men vs. bisexual, vs. transgender).^16^ In this present study, we sought to evaluate the sleep of TGNB individuals specifically to provide insight into how health care workers should approach working with these populations to reduce the health disparities and improve TGNB health.

Second, we found that 15% of TGNB participants reported struggles with anxiety and/or depression, which hindered their ability to fall asleep and remain asleep. Sleep for these individuals showed improvement through the use of pharmaceutical treatment of mental illness, particularly psychiatric medication. Of note, however, one of these six participants demonstrated that anxiety leading to poor sleep quality may be a risk factor for overeating, and overall poor eating habits.

Encouragingly, a consistently noted outcome or by-product in interventions aimed at improving mental health is improved sleep^39^; furthermore, interventions to improve sleep have also demonstrated improvements in mental health.^40,41^ Intervention strategies should likewise target mental health among TGNB individuals as a proven therapeutic measure to reduce depression, anxiety, and symptoms of post-traumatic stress disorder, which will, in turn, help to improve sleep health overall for this population. To the best of our knowledge, no existing sleep health interventions have focused on TGNB populations specifically, and this warrants further investigation.

Third, we found that a significant number (35%) of participants reported that their gender identity contributed to disruptive sleep, including feelings of gender dysphoria, self-consciousness about their physical body, stress and anxiety of identifying as transgender, and the process of gender transition (hormonal therapy and gender-affirming surgery). Gender dysphoria is defined as distress that may accompany the gender incongruence between one's experienced or expressed gender and one's assigned sex, often causing one to suffer from psychosocial stress.^42,43^

Five participants described how the undesired presence of breasts negatively affected their sleep quality, and one participant even reported breast binding as a coping mechanism for their gender dysphoria. Increasing evidence has demonstrated that social gender transition (using a different name or changing their style of clothing to that which is stereotypically associated with another gender) and medical gender transition (hormonal therapy and gender-affirming surgery) reduce gender dysphoria, leading to reduced stress and improvement in gender dysphoria,^44,45^ which could in turn lead to improvement in sleep health.

However, as reported by one of the participants, unsatisfactory gender-affirming surgery can in fact exacerbate rather than alleviate feelings of gender dysphoria. The contributory role of gender identity in the sleep health of TGNB individuals has thus far remained unstudied, and future research is still needed to further elucidate the nuances of gender dysphoria and existing coping mechanisms to sleep.

Lastly, the majority (60%) of participants in our study engaged in some form of coping mechanism to improve sleep quality with 32.5% of participants reporting the use of prescription or OTC sleep aid medication and 17.5% of participants reporting inconsistent use of marijuana as a sleep aid. While prescription medications have been used historically for the treatment of insomnia, the misuse of prescription drugs has been linked to negative outcomes, including dependence, overdose, and adverse drug interactions,^38,46,47^ especially when taken in conjunction with alcohol and/or other drugs.^48^

In our study, participants reported the use of such prescription pharmaceuticals as sedative hypnotics (triazolam and clonazepam), atypical antipsychotics (Seroquel), and antihypertensive medication (prasozin), each having well-established serious side effect profiles if misused. For example, the potential consequences of sedative use in conjunction with alcohol and opioids can lead to multiple health problems, including respiratory failure, seizures, coma, and death.^47,48^

Although our study asked participants the question, “Are there any medications or supplements that you take to help yourself sleep?” without specifying whether taken with or without a doctor's prescription, it may be appropriate to consider nonmedical use of prescription drugs (NMUPD) due to the robust association between mental distress and NMUPD.^49^ Prior work suggests that transgender adults may be more vulnerable to emotional distress, stigmatization, and gender identity-related discrimination,^33,50,51^ and have relatively high rates of both substance use^52,53^ and psychiatric symptoms.^50^

Furthermore, more refined work is needed to better understand the use of prescription medication among TGNB individuals. Future studies should further probe the use of specific medication classes taken as sleep aids, and intervention efforts should be geared toward assessing for NMUPD and treating underlying mental illness to improve sleep health of TGNB individuals.

## Study Limitations

While the novel qualitative data in this study seek to highlight the voices and insights of TGNB individuals about their own sleep health experiences, we acknowledge several limitations in this study.

First, the discussion and insights examined are derived from the unique experiences and narratives of the participants in the study and therefore cannot be used to generalize for all TGNB-identifying individuals. Second, the experiences and occurrences as described by participants may be unique to New York City alone, and cannot be generalized as a phenomenon occurring in other cities. Third, our sample size, while common for qualitative research inquiry, was modest and therefore cannot be generalized toward a larger population. Moreover, the sample was self-selected, and participants' prior knowledge of related studies may have influenced the content and quality of responses.

Despite these limitations, the strengths of the study include exploring factors related to sleep among TGNB individuals, which can assist in guiding future research directions.

## Conclusion

In conclusion, the main findings of our study showed that the sleep health of TGNB individuals was affected by a self-reported presence of mental illness, depression, or anxiety as well as gender-related concerns, including gender dysphoria. Furthermore, our study demonstrated that treated mental illness led to self-reported improvement in sleep in contrast to untreated mental illness. This research also suggests that many TGNB individuals cope with poor sleep quality through both pharmaceutical and nonpharmaceutical therapies. Our study findings may have implications for both the clinical management and broader intervention strategies tailored for this target population. We also provide a platform for the production of refined future qualitative and quantitative studies with this key population.

## Abbreviations Used

MSM: men who have sex with men
NMUPD: nonmedical use of prescription drugs
OTC: over-the-counter
TGNB: transgender and gender nonbinary

## References

[1] Institute of Medicine Committee on Sleep Medicine and Research; Colten HR, Altevogt BM, eds. The National Academies Collection: Reports Funded by National Institutes of Health. Sleep Disorders and Sleep Deprivation: An Unmet Public Health Problem. Washington, DC: National Academies Press (US) National Academy of Sciences, 2006 20669438

[2] Ayas NT, White DP, Manson JE, et al. A prospective study of sleep duration and coronary heart disease in women. Arch Intern Med. 2003;163:205–209 12546611 10.1001/archinte.163.2.205

[3] Buxton OM, Marcelli E Short and long sleep are positively associated with obesity, diabetes, hypertension, and cardiovascular disease among adults in the United States. Soc Sci Med. 2010;71:1027–1036 20621406 10.1016/j.socscimed.2010.05.041

[4] Cappuccio FP, D'Elia L, Strazzullo P, Miller MA Sleep duration and all-cause mortality: a systematic review and meta-analysis of prospective studies. Sleep. 2010;33:585–592 20469800 10.1093/sleep/33.5.585 PMC2864873

[5] Gallicchio L, Kalesan B Sleep duration and mortality: a systematic review and meta-analysis. J Sleep Res. 2009;18:148–158 19645960 10.1111/j.1365-2869.2008.00732.x

[6] Gottlieb DJ, Punjabi NM, Newman AB, et al. Association of sleep time with diabetes mellitus and impaired glucose tolerance. Arch Intern Med. 2005;165:863–867 15851636 10.1001/archinte.165.8.863

[7] Grandner MA, Chakravorty S, Perlis ML, et al. Habitual sleep duration associated with self-reported and objectively determined cardiometabolic risk factors. Sleep Med. 2014;15:42–50 24333222 10.1016/j.sleep.2013.09.012 PMC3947242

[8] Liu Y, Wheaton AG, Chapman DP, Croft JB. Sleep duration and chronic diseases among U.S adults age 45 years and older: evidence from the 2010 Behavioral Risk Factor Surveillance System. Sleep. 2013;36:1421–1427 24082301 10.5665/sleep.3028 PMC3773191

[9] Nagai M, Hoshide S, Kario K Sleep duration as a risk factor for cardiovascular disease- a review of the recent literature. Cur Cardiol Rev. 2010;6:54–61 10.2174/157340310790231635 PMC2845795 21286279

[10] Sabanayagam C, Shankar A Sleep duration and cardiovascular disease: results from the National Health Interview Survey. Sleep. 2010;33:1037–1042 20815184 10.1093/sleep/33.8.1037 PMC2910533

[11] van den Berg JF, Luijendijk HJ, Tulen JH, et al. Sleep in depression and anxiety disorders: a population-based study of elderly persons. J Clin Psychiatry. 2009;70:1105–1113 19607762 10.4088/JCP.08m04448

[12] von Ruesten A, Weikert C, Fietze I, Boeing H Association of sleep duration with chronic diseases in the European Prospective Investigation into Cancer and Nutrition (EPIC)-Potsdam study. PLoS One. 2012;7:e30972 22295122 10.1371/journal.pone.0030972 PMC3266295

[13] Blosnich JR, Farmer GW, Lee JG, et al. Health inequalities among sexual minority adults: evidence from ten U.S states, 2010. Am J Prev Med. 2014;46:337–349 24650836 10.1016/j.amepre.2013.11.010 PMC4102129

[14] Chen JH, Shiu CS Sexual orientation and sleep in the U.S.: a national profile. Am J Prev Med. 2017;52:433–442 28062273 10.1016/j.amepre.2016.10.039

[15] Cunningham TJ, Xu F, Town M. Prevalence of five health-related behaviors for chronic disease prevention among sexual and gender minority adults–25 U.S states and Guam, 2016. MMWR Morb Mortal Wkly Rep. 2018;67:888–893 30114006 10.15585/mmwr.mm6732a4 PMC6095649

[16] Dai H, Hao J Sleep deprivation and chronic health conditions among sexual minority adults. Behav Sleep Med. 2017:1–15 10.1080/15402002.2017.1342166 28657361

[17] Downing MJ Jr., Houang ST, Scheinmann R, et al. Engagement in care, psychological distress, and resilience are associated with sleep quality among HIV-positive gay, bisexual, and other men who have sex with men. Sleep Health. 2016;2:322–329 28191491 10.1016/j.sleh.2016.08.002 PMC5300085

[18] Duncan DT, Goedel WC, Mayer KH, et al. Poor sleep health and its association with mental health, substance use, and condomless anal intercourse among gay, bisexual, and other men who have sex with men. Sleep Health. 2016;2:316–321 29073390 10.1016/j.sleh.2016.07.003 PMC5689458

[19] Duncan DT, Kanchi R, Tantay L, et al. Disparities in sleep problems by sexual orientation among New York City adults: an analysis of the New York City Health and Nutrition Examination Survey, 2013–2014. J Urban Health. 2018;95:781–786 29987769 10.1007/s11524-018-0268-0 PMC6286285

[20] Fredriksen-Goldsen KI, Kim HJ, Barkan SE, et al. Health disparities among lesbian, gay, and bisexual older adults: results from a population-based study. Am J Public Health. 2013;103:1802–1809 23763391 10.2105/AJPH.2012.301110 PMC3770805

[21] Fricke J, Sironi M Dimensions of sexual orientation and sleep disturbance among young adults. Prev Med Rep. 2017;8:18–24 28831369 10.1016/j.pmedr.2017.07.008 PMC5555087

[22] Galinsky AM, Ward BW, Joestl SS, Dahlhamer JM Sleep duration, sleep quality, and sexual orientation: findings from the 2013–2015 National Health Interview Survey. Sleep Health. 2018;4:56–62 29332681 10.1016/j.sleh.2017.10.004 PMC6317849

[23] Li P, Huang Y, Guo L, et al. Is sexual minority status associated with poor sleep quality among adolescents? Analysis of a national cross-sectional survey in Chinese adolescents. BMJ Open. 2017;7:e017067 10.1136/bmjopen-2017-017067 PMC5770949 29282258

[24] Martin-Storey A, Prickett KC, Crosnoe R Disparities in sleep duration and restedness among same- and different-sex couples: findings from the American Time Use Survey. Sleep. 2018;41:1–11 10.1093/sleep/zsy090 PMC6093344 29726972

[25] Millar BM, Parsons JT, Redline S, Duncan DT What's sleep got to do with it?: Sleep health and sexual risk-taking among men who have sex with men. AIDS Behav. 2018;23:572–579 10.1007/s10461-018-2288-x 30267366

[26] Patterson CJ, Tate DP, Sumontha J, Xu R Sleep difficulties among sexual minority adults: associations with family relationship problems. Psychol Sex Orientat Gender Divers. 2018;5:109–116

[27] Duncan DT, Park SH, Goedel WC, et al. Perceived neighborhood safety is associated with poor sleep health among gay, bisexual, and other men who have sex with men in Paris, France. J Urban Health. 2017;94:399–407 28439769 10.1007/s11524-017-0148-z PMC5481216

[28] Duncan DT, Hyun Park S, Al-Ajlouni YA, et al. Association of financial hardship with poor sleep health outcomes among men who have sex with men. SSM Popul Health. 2017;3:594–599 29349248 10.1016/j.ssmph.2017.07.006 PMC5769031

[29] Auer MK, Liedl A, Fuss J, et al. High impact of sleeping problems on quality of life in transgender individuals: a cross-sectional multicenter study. PLoS One. 2017;12:e0171640 28199359 10.1371/journal.pone.0171640 PMC5310898

[30] Kunzel HE, Murck H, Stalla GK, Steiger A Changes in the sleep electroencephalogram (EEG) during male to female transgender therapy. Psychoneuroendocrinology. 2011;36:1005–1009 21273005 10.1016/j.psyneuen.2010.12.014

[31] Bockting WO, Miner MH, Swinburne Romine RE, et al. Stigma, mental health, and resilience in an online sample of the US transgender population. Am J Public Health. 2013;103:943–951 23488522 10.2105/AJPH.2013.301241 PMC3698807

[32] Bornstein DR, Fawcett J, Sullivan M, et al. Understanding the experiences of lesbian, bisexual and trans survivors of domestic violence: a qualitative study. J Homosex. 2006;51:159–181 16893830 10.1300/J082v51n01_08

[33] Clements-Nolle K, Marx R, Guzman R, Katz M HIV prevalence, risk behaviors, health care use, and mental health status of transgender persons: implications for public health intervention. Am J Public Health. 2001;91:915–921 11392934 10.2105/ajph.91.6.915 PMC1446468

[34] Schneider M. Values and Preferences of Transgender People: A Qualitative Study to Inform the Consolidated Guidelines on HIV Prevention, Diagnosis, Treatment and Care for Key Populations Development Process. Geneva: World Health Organization, 2014 25996019

[35] Poteat T, German D, Kerrigan D Managing uncertainty: a grounded theory of stigma in transgender health care encounters. Soc Sci Med. 2013;84:22–29 23517700 10.1016/j.socscimed.2013.02.019

[36] Whyte WF, Greenwood DJ, Lazes P. Participatory Action Research, through practice to science in social research. In: Participatory Action Research. (Whyte WF; ed). Newbury Park, CA: Sage Publications, 1991, pp. 19–55

[37] Xavier J, Bradford J, Hendricks M, et al. Transgender health care access in Virginia: a qualitative study. Int J Transgenderism. 2013;14:3–17

[38] Boyatzis RE. Transforming qualitative information: thematic analysis and code development. Thousand Oaks, CA: Sage Publications, Inc., 1998

[39] Yon A, Scogin F, DiNapoli EA, et al. Do manualized treatments for depression reduce insomnia symptoms? J Clin Psychol. 2014;70:616–630 24596077 10.1002/jclp.22062

[40] Franzen PL, Buysse DJ Sleep disturbances and depression: risk relationships for subsequent depression and therapeutic implications. Dialogues Clin Neurosci. 2008;10:473–481 19170404 10.31887/DCNS.2008.10.4/plfranzen PMC3108260

[41] Howland RH. Sleep interventions for the treatment of depression. J Psychosoc Nurs Ment Health Serv. 2011;49:17–20 10.3928/02793695-20101208-01 21175118

[42] Drescher J, Cohen-Kettenis P, Winter S Minding the body: situating gender identity diagnoses in the ICD-11. Int Rev Psychiatry. 2012;24:568–577 23244612 10.3109/09540261.2012.741575

[43] Vahia VN. Diagnostic and statistical manual of mental disorders 5: a quick glance. Indian J Psychiatry. 2013;55:220–223 24082241 10.4103/0019-5545.117131 PMC3777342

[44] de Vries AL, McGuire JK, Steensma TD, et al. Young adult psychological outcome after puberty suppression and gender reassignment. Pediatrics. 2014;134:696–704 25201798 10.1542/peds.2013-2958

[45] Guss C, Shumer D, Katz-Wise SL Transgender and gender nonconforming adolescent care: psychosocial and medical considerations. Curr Opin Pediatr. 2015;27:421–426 26087416 10.1097/MOP.0000000000000240 PMC4522917

[46] Substance Abuse and Mental Health Services Administration. Results from the 2010 National Survey on Drug Use and Health: Summary of National Findings, NSDUH Series H-41, HHS Publication No. (SMA) 11-4658. Rockville, MD: Substance Abuse and Mental Health Services Administration, 2011

[47] Benotsch EG, Zimmerman R, Cathers L, et al. Non-medical use of prescription drugs, polysubstance use, and mental health in transgender adults. Drug Alcohol Depend. 2013;132:391–394 23510637 10.1016/j.drugalcdep.2013.02.027

[48] National Institute on Alcohol Abuse and Alcoholism. Harmful interactions: Mixing alcohol with medicines. Bethesda, MD: National Institutes of Health, U.S. Department of Health and Human Services (DHHS), revised 2014 (NIH Publication No. 13-5329). Available at http://pubs.niaa.nih.gov/publications/Medicine/medicine.htm Accessed 1010, 2018

[49] Zullig KJ, Divin AL The association between non-medical prescription drug use, depressive symptoms, and suicidality among college students. Addict Behav. 2012;37:890–899 22541802 10.1016/j.addbeh.2012.02.008

[50] Haas AP, Eliason M, Mays VM, et al. Suicide and suicide risk in lesbian, gay, bisexual, and transgender populations: review and recommendations. J Homosex. 2011;58:10–51 21213174 10.1080/00918369.2011.534038 PMC3662085

[51] Nemoto T, Bodeker B, Iwamoto M Social support, exposure to violence and transphobia, and correlates of depression among male-to-female transgender women with a history of sex work. Am J Public Health. 2011;101:1980–1988 21493940 10.2105/AJPH.2010.197285 PMC3222349

[52] Herbst JH, Jacobs ED, Finlayson TJ, et al. Estimating HIV prevalence and risk behaviors of transgender persons in the United States: a systematic review. AIDS Behav. 2008;12:1–17 17694429 10.1007/s10461-007-9299-3

[53] Lawrence AA. Gender identity disorders in adults: diagnosis and treatment. In: Handbook of Sexual and Gender Identity Disorders. (Rowland DL, Incrocci L; eds). New York: Wiley, 2008, pp. 423–456

